# Supplementary material for: Association between Interleukin-1 Polymorphisms and Susceptibility to Dental Peri-Implant Disease: A Meta-Analysis
Source: Pathogens. 2021 Dec 9;10(12):1600. doi: 10.3390/pathogens10121600 (PMC8707532; doi:10.3390/pathogens10121600)
Supplement: Supplementary file 1 [file pathogens-10-01600-s001.zip › pathogens-1483303-supplementary.pdf]

Table S1. Characteristics of the studies included in the meta-analysis.

First author, publication year	Country	Ethnicity	No. of Cases	No. of Controls	Polymorphism (s): *p*-value of HWE in control groups	Outcome	Matched factors* (cases vs. controls)	Quality score
Rogers, 2002[27]	Australia	Caucasian	19	31	*IL-1A (-889)*: 0.380, *IL-1B (+3953)*: 0.694, Composite genotype of *IL-1A (-889)* and *IL-1B (+3953)*	Implant failure	NR	7
Shimpuku, 2003[28]	Japan	Asian	17	22	*IL-1A (-889)*: 0.639, *IL-1B (-511)*: 0.338, *IL-1B (+3954)*: 0.823	Marginal bone loss	Age, sex, and smoking	7
Campos, 2005 [29]	Brazil	Mixed	28	34	*IL-1A (-889)*: 0.157, *IL-1B (-511)*: 0.790, *IL-1B (+3953)*: 0.558, *IL-1RN (VNTR)*: 0.863, Composite genotype of *IL-1A (-889)* and *IL-1B (+3953)*	Implant failure	Age, sex, and smoking	7
Laine, 2006[30]	Sweden	Caucasian	71	49	*IL-1A (-889)*: 0.102, *IL-1B (-511)*: 0.437, *IL-1B (+3954)*: 0.783, Composite genotype of *IL-1A (-889)* and *IL-1B (+3954)*	Peri-implantitis	Age	7
Lachmann, 2007[31]	German	Caucasian	11	18	Composite genotype of *IL-1A (-889)* and *IL-1B (+3954)*	Peri-implantitis	NR	6
Lin, 2007[32]	China	Asian	29	30	*IL-1B (-511)*: 0.955 *IL-1B (+3954)*: 0.850	Marginal bone loss	Age, sex, and smoking	8
Montes, 2009[33]	Brazil	Mixed	90	176	*IL-1B (+3954)*: 0.779	Implant failure	Age, sex, and smoking	7
Dirschnabel, 2011[34]	Brazil	Mixed	92	185	*IL-1B (-511)*: 0.994	Implant failure	Age, sex, and smoking	8
Hamdy, 2011[35]	Egypt	Caucasian	25	25	Composite genotype of *IL-1A (-889)* and *IL-1B (+3954)*	Peri-implantitis	Age, sex, and smoking	8
Melo, 2012[36]	Brazil	Mixed	16	31	*IL-1A (-889)*: 0.976, *IL-1B (-511)*: 0.213, *IL-1B (+3954)*: 0.380	Peri-implantitis	Age, sex, and smoking	7
Vaz, 2012[37]	Portugal	Caucasian	55	100	Composite genotype of *IL-1A (-889)* and *IL-1B (+3953)*	Implant failure or biological complications	NR	6
Jacobi-Gresser, 2013[38]	German	Caucasian	41	68	*IL-1A (-889)*: 0.606 *IL-1B (+3953)*: 0.163	Implant failure	Age and smoking	8
Cosyn, 2016 [15]	Belgium	Caucasian	14	14	*IL-1A (-889)*: 0.512, *IL-1B (-511)*: 0.803, *IL-1B (+3954)*: 0.889	Implant failure	Age, sex, and smoking	8
Petkovic-Curcin, 2017 [39]	Serbia	Caucasian	34	64	*IL-1RN (VNTR)*: 0.516	Peri-implantitis	Age and sex	8
Agrawal, 2021 [40]	India	Asian	68	63	*IL-1A (-889)*: 0.730, *IL-1B (-511)*: 0.249, *IL-1B (+3954)*: 0.479	Marginal bone loss	NR	7
Saremi, 2021 [41]	Iran	Caucasian	50	89	*IL-1B (+3954)*: 0.699	Peri-implantitis	Age and sex	9

NR: Not reported. * Age, sex, and smoking.

Table S2. Subgroup analysis of the association between alleles, genotypes of *IL-1A (-889)* polymorphism and the risk of dental PID.

Variable (N)	T *vs.* C	TT *vs.* CC	TC *vs.* CC	TT + TC *vs.* CC	TT *vs.* CC + TC
	OR (95%CI), *p*, I^2	OR (95%CI), *p*, I^2	OR (95%CI), *p*, I^2	OR (95%CI), *p*, I^2	OR (95%CI), *p*, I^2
All (8)	1.19 (0.92, 1.55), 0.19, 0%	1..18 (0.62, 2.25), 0.61, 0%	1.45 (0.97, 2.16), 0.07, 0%	1.43 (0.98, 2.10), 0.07, 0%	1.02 (0.64, 1.63), 0.94, 0%
Ethnicity					
Caucasian (4)	1.35 (0.95, 1.93), 0.09, 29%	1.27 (0.47, 3.40), 0.63, 25%	1.79 (1.10, 2.89), **0.02**, 0%	1.73 (1.08, 2.76), **0.02**, 0%	0.95 (0.37, 2.49), 0.92, 15%
Asian (2)	1.10 (0.66, 1.82), 0.71, 0%	1.19 (0.40, 3.55), 0.75, 0%	0.76 (0.28, 2.10), 0.60, 0%	0.93 (0.36, 2.39), 0.88, 0%	1.23 (0.63, 2.40), 0.54, 0%
Mixed (2)	0.90 (0.48, 1.68), 0.74, 0%	1.02 (0.27, 3.90), 0.98, 0%	1.08 (0.38, 3.09), 0.88, 0%	1.02 (0.39, 2.65), 0.96, 0%	0.75 (0.29, 1.91), 0.54, 0%
Outcome					
Peri-implantitis (2)	0.98 (0.60, 1.58), 0.92, 0%	0.67 (0.14, 3.29), 0.62, 0%	1.36 (0.65, 2.88), 0.41, 0%	1.26 (0.60, 2.63), 0.54, 0%	0.55 (0.20, 1.54), 0.26, 0%
Implant failure (4)	1.43 (0.96, 2.13), 0.08, 24%	1.42 (0.57, 3.55), 0.45, 7%	1.80 (1.05, 3.09), **0.03**, 0%	1.73 (1.03, 2.89), **0.04**, 0%	1.16 (0.47, 2.81), 0.75, 0%
Marginal bone loss (2)	1.10 (0.66, 1.82), 0.71, 0%	1.19 (0.40, 3.55), 0.75, 0%	0.76 (0.28, 2.10), 0.60, 0%	0.93 (0.36, 2.39), 0.88, 0%	1.23 (0.63, 2.40), 0.54, 0%
Sample size					
>100 (3)	1.20 (0.87, 1.66), 0.26, 0%	1.08 (0.46, 2.54), 0.86, 0%	1.45 (0.87, 2.40), 0.15, 0%	1.44 (0.88, 2.35), 0.14, 0%	1.09 (0.60, 1.98), 0.77, 0%
≤100 (5)	1.16 (0.74, 1.83), 0.51, 1%	1.33 (0.50, 3.51), 0.57, 0%	1.45 (0.75, 2.78), 0.27, 0%	1.41 (0.77, 2.61), 0.27, 0%	0.91 (0.42, 1.96), 0.80, 0%

Bold number shows the association is statistically significant ($p < 0.05$).

Table S3. Subgroup analysis of the association between alleles, genotypes of *IL-1B (-511)* polymorphism and the risk of dental PID.

Variable (N)	T *vs.* C	TT *vs.* CC	TC *vs.* CC	TT + TC *vs.* CC	TT *vs.* CC + TC
	OR (95%CI), *p*, I^2	OR (95%CI), *p*, I^2	OR (95%CI), *p*, I^2	OR (95%CI), *p*, I^2	OR (95%CI), *p*, I^2
All (8)	1.10 (0.69, 1.75), 0.70, 75%	1.20 (0.59, 2.42), 0.61, 54%	0.72 (0.52, 1.01), 0.06, 18%	0.84 (0.61, 1.14), 0.25, 48%	1.45 (1.00, 2.09), 0.05, 42%
Ethnicity					
Caucasian (2)	0.95 (0.59, 1.53), 0.83, 0%	1.04 (0.36, 2.94), 0.95, 0%	0.77 (0.37, 1.60), 0.48, 0%	0.83 (0.41, 1.66), 0.59, 0%	1.14 (0.46, 2.81), 0.78, 0%
Asian (3)	1.55 (0.36, 6.73), 0.56, 92%	1.49 (0.18, 12.22), 0.71, 84%	0.47 (0.25, 0.86), **0.02**, 19%	0.85 (0.23, 3.16), 0.81, 75%	1.97 (0.38, 10.20), 0.42, 82%
Mixed (3)	1.12 (0.83, 1.52), 0.46, 8%	1.45 (0.80, 2.64), 0.22, 0%	0.91 (0.57, 1.46), 0.70, 38%	1.05 (0.68, 1.62), 0.83, 28%	1.55 (0.90, 2.64), 0.11, 0%
Outcome					
Peri-implantitis (2)	0.96 (0.59, 1.54), 0.85, 0%	1.35 (0.47, 3.90), 0.58, 0%	0.90 (0.46, 1.77), 0.77, 0%	0.97 (0.51, 1.86), 0.94, 0%	1.41 (0.53, 3.76), 0.49, 0%
Implant failure (3)	1.12 (0.82, 1.52), 0.47, 12%	1.33 (0.73, 2.41), 0.35, 0%	0.85 (0.52, 1.39), 0.52, 32%	0.98 (0.63, 1.54), 0.94, 28%	1.43 (0.85, 2.41), 0.18, 0%
Marginal bone loss (3)	1.55 (0.36, 6.73), 0.56, 92%	1.49 (0.18, 12.22), 0.71, 84%	0.47 (0.25, 0.86), **0.02**, 19%	0.85 (0.23, 3.16), 0.81, 75%	1.97 (0.38, 10.20), 82%
Sample size					
>100 (3)	0.85 (0.45, 1.60), 0.61, 83%	0.82 (0.24, 2.84), 0.76, 78%	0.67 (0.33, 1.36), 0.27, 67%	0.70 (0.30, 1.61), 0.40, 79%	1.06 (0.46, 2.43), 0.89, 60%
≤100 (5)	1.36 (0.66, 2.80), 0.41, 70%	1.68 (0.82, 3.44), 0.16, 16%	0.72 (0.38, 1.36), 0.32, 0%	0.99 (0.55, 1.76), 0.97, 0%	2.06 (1.13, 3.75), **0.02**, 23%

Bold number shows the association is statistically significant ($p < 0.05$).

Table S4. Subgroup analysis of the association between alleles, genotypes of *IL-1B (+3954)* polymorphism and the risk of dental PID.

Variable (N)	T *vs.* C	TT *vs.* CC	TC *vs.* CC	TT + TC *vs.* CC	TT *vs.* CC + TC
	OR (95%CI), *p*, I^2	OR (95%CI), *p*, I^2	OR (95%CI), *p*, I^2	OR (95%CI), *p*, I^2	OR (95%CI), *p*, I^2
All (7)	2.04 (1.02, 4.08), **0.04**, 73%	1.73 (0.45, 6.58), 0.42, 52%	1.68 (1.18, 2.39), **0.004**, 49%	2.27 (1.11, 4.64), **0.03**, 65%	1.19 (0.58, 2.45), 0.63, 42%
Ethnicity					
Caucasian (3)	3.61 (0.66, 19.75), 0.14, 88%	3.76 (0.27, 52.13), 0.32, 71%	2.91 (0.81, 10.40), 0.10, 71%	3.59 (0.75, 17.28), 0.11, 82%	2.78 (0.25, 30.80), 0.40, 66%
Asian (2)	2.33 (0.67, 8.05), 0.18, 34%	Not estimable	2.45 (0.69, 8.74), 0.17, 39%	2.45 (0.69, 8.74), 0.17, 39%	Not estimable
Mixed (2)	1.13 (0.77, 1.67), 0.52, 42%	0.85 (0.26, 2.80), 0.78, 28%	1.36 (0.84, 2.21), 0.21, 47%	1.25 (0.79, 1.99), 0.34, 16%	0.69 (0.21, 2.26), 0.54, 0%
Outcome					
Peri-implantitis (3)	2.12 (0.67, 6.66), 0.20, 84%	2.20 (0.31, 15.78), 0.43, 63%	2.26 (0.91, 5.63), 0.08, 59%	2.43 (0.78, 7.58), 0.13, 76%	1.58 (0.27, 9.29), 0.61, 59%
Implant failure (2)	3.16 (0.22, 45.01), 0.40, 84%	1.66 (0.09, 30.16), 0.73, 64%	2.97 (0.30, 29.51), 0.35, 74%	3.47 (0.24, 50.45), 0.36, 81%	0.87 (0.26, 2.92), 0.82, 49%
Marginal bone loss (2)	2.33 (0.67, 8.05), 0.18, 34%	Not estimable	2.45 (0.69, 8.74), 0.17, 39%	2.45 (0.69, 8.74), 0.17, 39%	Not estimable
Sample size					
>100 (3)	1.25 (0.92, 1.71), 0.15, 86%	1.10 (0.48, 2.54), 0.82, 65%	1.41 (0.95, 2.09), 0.09, 61%	1.40 (0.96, 2.04), 0.08, 78%	1.00 (0.44, 2.28), 0.99, 62%
≤100 (4)	2.88 (1.49, 5.58), **0.002**, 35%	4.07 (0.80, 20.69), 0.09, 0%	3.58 (1.55, 8.28), **0.003**, 5%	4.80 (1.95, 11.82), **0.0006**, 0%	2.18 (0.47, 10.02), 0.32, 0%

Bold number shows the association is statistically significant ($p < 0.05$).

Table S5. Meta-regression analysis of the association between alleles and genotypes of *IL-1A (-889)*, *IL-1B (-511)*, and *IL-1B (+3954)* polymorphisms and the risk of dental PID.

Variable		*IL-1A (-889)*				
		T *vs*. C	TT *vs*. CC	TC *vs*. CC	TT + CT *vs*. CC	TT *vs*. CC + CT
Year of publication	R	0.375	0.330	0.293	0.375	0.237
	Adjusted R^2	-0.003	-.040	-0.067	-0.002	-0.101
	P-value	0.361	0.425	0.482	0.359	0.571
Sample size	R	0.352	0.496	0.384	0.385	0.496
	Adjusted R^2	-0.022	0.121	0.006	0.006	0.120
	P-value	0.393	0.211	0.347	0.347	0.211
Ethnicity	R	0.468	0.297	0.350	0.450	0.244
	Adjusted R^2	0.088	-0.064	-0.024	0.070	-0.097
	P-value	0.243	0.475	0.396	0.263	0.561
Outcome	R	0.109	0.164	0.453	0.156	0.363
	Adjusted R^2	-0.153	-0.135	0.073	-0.138	-0.013
	P-value	0.798	0.698	0.259	0.713	0.376
Variable		*IL-1B (-511)*				
		T *vs*. C	TT *vs*. CC	TC *vs*. CC	TT + CT *vs*. CC	TT *vs*. CC + CT
Year of publication	R	0.521	0.651	0.222	0.556	0.683
	Adjusted R^2	0.150	0.328	-0.109	0.194	0.378
	P-value	0.185	0.080	0.597	0.152	0.062
Sample size	R	0.170	0.222	0.081	0.092	0.255
	Adjusted R^2	-0.133	-0.109	-0.159	-0.157	-0.091
	P-value	0.687	0.598	0.848	0.828	0.542
Ethnicity	R	0.050	0.047	0.257	0.193	0.007
	Adjusted R^2	-0.164	-0.164	-0.090	-0.123	-0.167
	P-value	0.907	0.911	0.539	0.648	0.988
Outcome	R	0.520	0.422	0.400	0.106	0.487
	Adjusted R^2	0.149	0.042	0.020	-0.154	0.110
	P-value	0.186	0.297	0.326	0.803	0.221
Variable		*IL-1B (+3954)*				
		T *vs*. C	TT *vs*. CC	TC *vs*. CC	TT + CT *vs*. CC	TT *vs*. CC + CT
Year of publication	R	0.639	0.948	0.567	0.588	0.911
	Adjusted R^2	0.290	0.866	0.186	0.214	0.773
	P-value	0.122	0.014	0.184	0.165	**0.032**
Sample size	R	0.366	0.190	0.435	0.404	0.086
	Adjusted R^2	-0.039	-0.285	0.027	-0.004	-0.324
	P-value	0.419	0.760	0.330	0.368	0.891
Ethnicity	R	0.532	0.567	0.427	0.472	0.532
	Adjusted R^2	0.140	0.095	0.019	0.068	0.044
	P-value	0.219	0.319	0.339	0.285	0.356
Outcome	R	0.004	0.164	0.025	0.008	0.248
	Adjusted R^2	-0.200	-0.297	-0.199	-0.200	-0.251
	P-value	0.992	0.792	0.958	0.986	0.687

Bold number shows the association is statistically significant ($p < 0.05$).

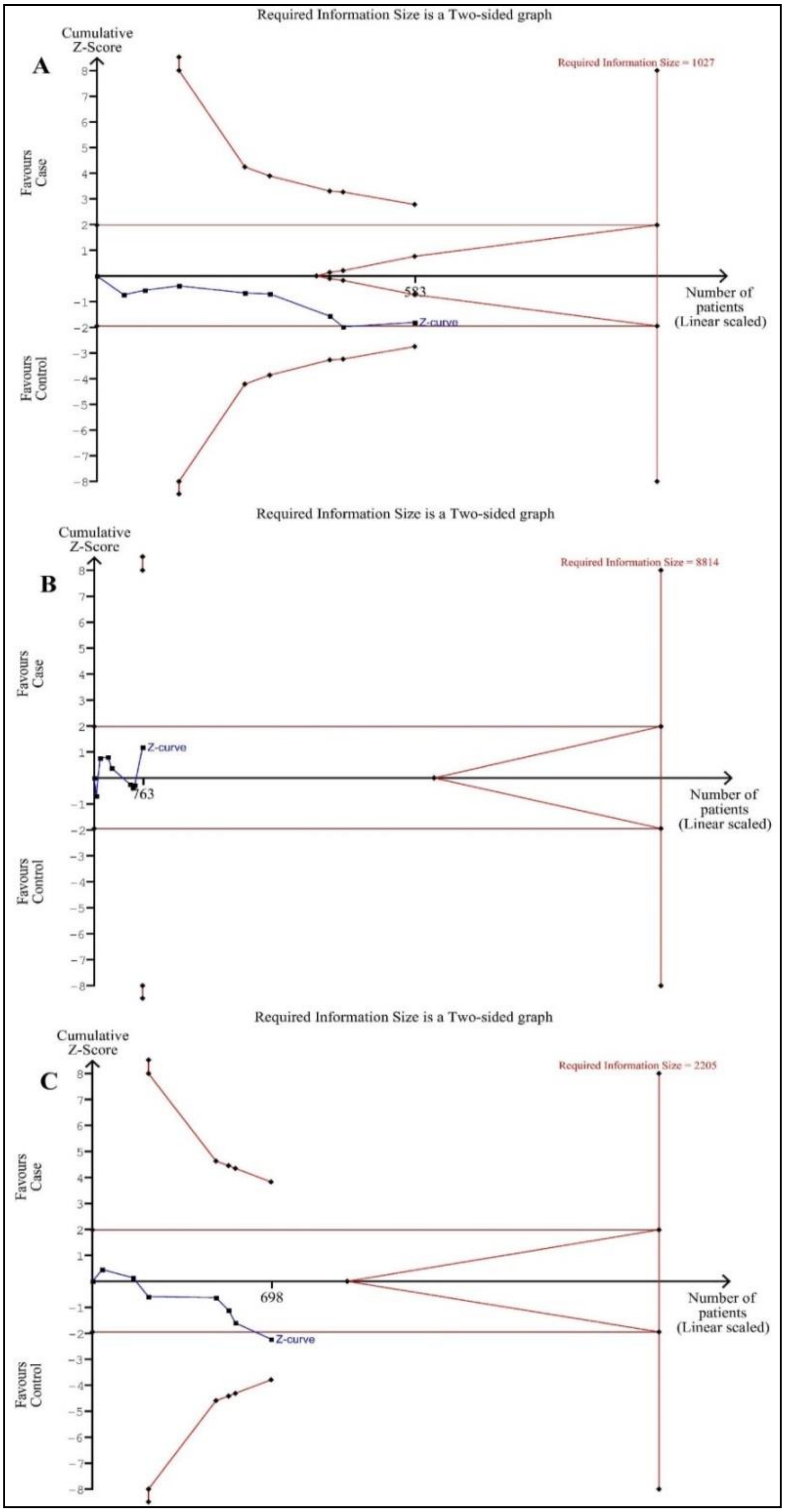

Figure S1. Trial sequential analysis of the association between genotypes of three polymorphisms and the risk of dental PID based on recessive model (TT + CT vs. CC). (A) IL-1A (-889) [D2 = 0%; RIS = 1027]; (B) IL-1B (-511) [D2 = 57%; RIS = 8814]; and (D) IL-1B (+3954) [D2 = 76%; RIS = 2205].

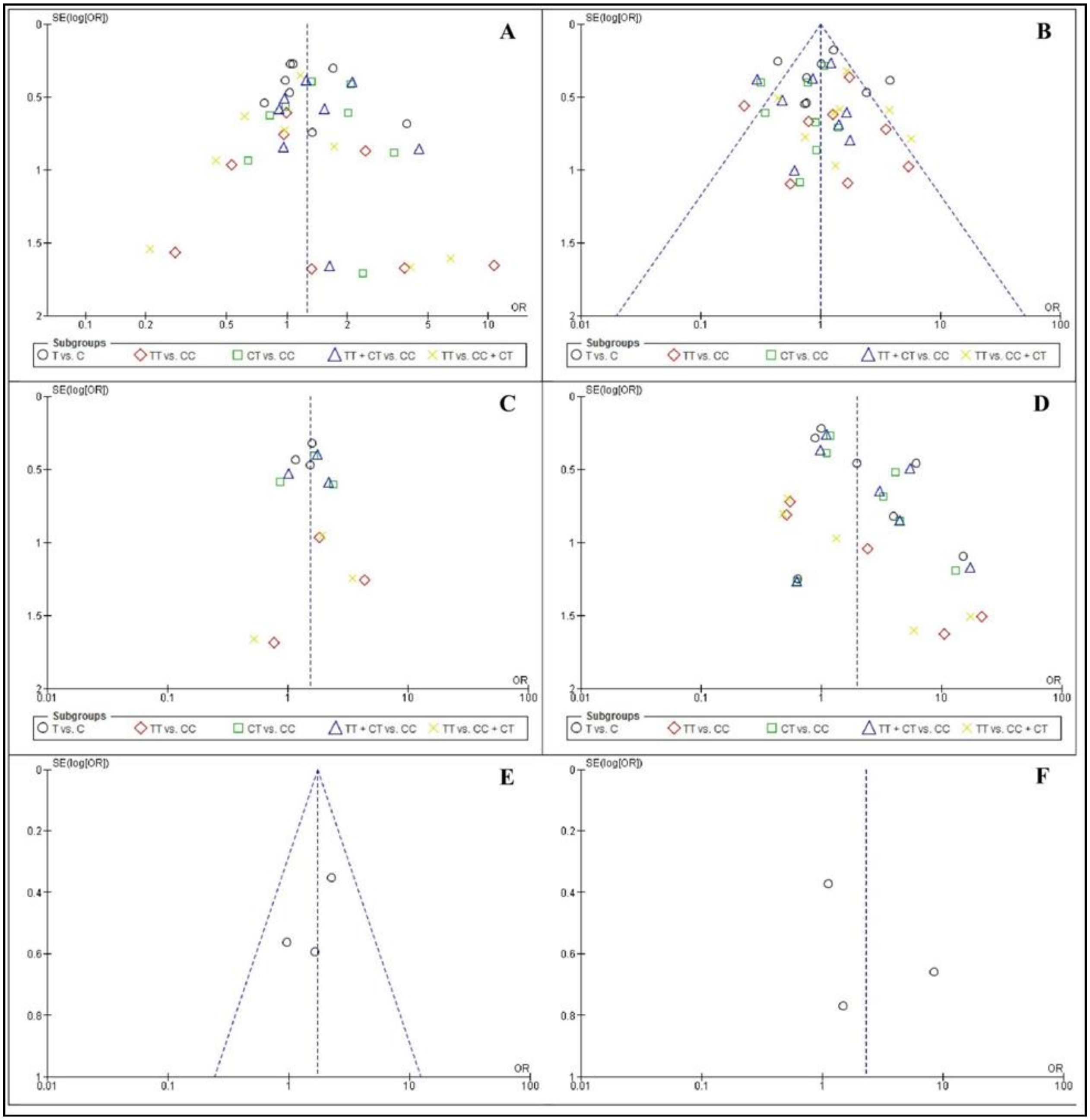

Figure S2. Funnel plot analysis of the association between alleles, genotypes of *IL-1* polymorphisms and the risk of dental PID. (A) *IL-1A (-889)*; (B) *IL-1B (-511)*; (C) *IL-1B (+3953)*; (D) *IL-1B (+3954)*; (E) composite genotype of *IL-1A (-889)*/*IL-1B (+3953)*; and (F) composite genotype of *IL-1A (-889)*/*IL-1B (+3954).*
